# Systemic metabolic benefits of 17α-estradiol are not exclusively mediated by ERα in glutamatergic or GABAergic neurons

**DOI:** 10.1007/s11357-024-01192-2

**Published:** 2024-05-22

**Authors:** Celine Camon, Mel Prescott, Christine Neyt, Caroline Decourt, Michael B. Stout, Rebecca E. Campbell, Michael Garratt

**Affiliations:** 1 Department of Anatomy, University of Otago, Dunedin, New Zealand; 2 Centre for Neuroendocrinology, University of Otago, Dunedin, New Zealand; 3 Department of Physiology, University of Otago, Dunedin, New Zealand; 4 Aging and Metabolism Research Program, Oklahoma Medical Research Foundation, Oklahoma City, OK USA

**Keywords:** Estrogens, Ageing, Sexual dimorphism, Glucose tolerance

## Abstract

17α-Estradiol (17αE2), a less-feminising enantiomer of 17β-estradiol, has been shown to prolong lifespan and improve metabolic health in a sex-specific manner in male, but not in female mice. Recent studies have demonstrated the pivotal role of estrogen receptor α (ERα) in mediating the effects of 17αE2 on metabolic health. However, the specific tissues and/or neuronal signalling pathways that 17αE2 acts through remain to be elucidated. ERα expression in glutamatergic and GABAergic neurons (principal excitatory and inhibitory neurons respectively) in the hypothalamus is essential for estradiol signalling. Therefore, we hypothesised that knocking out ERα from one of these neuronal populations would attenuate the established beneficial metabolic effects of 17αE2 in male mice exposed to a high fat diet. To test this hypothesis we used two established brain specific ERα KO models, targeting either glutamatergic or GABAergic neurons (Vglut2/Vgat-ERαKO). We show that both of these ERα KO models exhibit a strong reduction in ERα expression in the arcuate nucleus of the hypothalamus, a control centre for metabolic regulation. Deletion of ERα from GABAergic neurons significantly diminished the effect of 17αE2 on body weight relative to controls, although these animals still show metabolic benefits with 17αE2 treatment. The response to 17αE2 was unaffected by ERα deletion in glutamatergic neurons. Our results support a benefit of 17αE2 treatment in protection against metabolic dysfunction, but these effects do not depend on exclusive ERα expression in glutamatergic and GABAergic neurons and persist when ERα expression is strongly reduced in the arcuate nucleus of the hypothalamus.

## Introduction

Sex hormone signalling is altered during ageing due to the decline in circulating sex hormones (including estrogens) and the receptiveness of target tissues to these hormones [1]. Estrogen signalling is believed to serve a protective role in the ageing process, particularly in the context of altered metabolic function and obesity. While the administration of estrogens can reduce age-associated adiposity [2], their application can also increase the risk of some reproductive cancers in a subset of people and lead to feminisation in males [3], illustrating their unsuitability as long-term treatments for ageing [4]. Unravelling the sex-specific signalling pathways that underlie metabolic dysfunction in ageing and the beneficial responses to estrogens could lead to novel treatments that provide the benefits of sex hormones while mitigating negative consequences.

17α-Estradiol (17αE2), a naturally occurring enantiomer of 17β-estradiol (17β-E2), was recently shown to beneficially modulate health outcomes in male mice. The National Institute on Ageing Interventions Testing Program (ITP) reported that 17αE2 extends the median lifespan in male, but not in female mice when treatment is initiated at 10 months of age [5, 6] or later in life at 16 or 20 months of age [7]. Interestingly, 17αE2 treatment leads to lifespan extension to a similar degree as reported with calorie restriction [8] and rapamycin treatment [9] in male mice. 17αE2 treatment significantly reduces regional adiposity and improves several metabolic measures including glucose tolerance, insulin sensitivity and ectopic lipid deposition in obese and aged male mice [10–14]. 17αE2 treatment also has male-specific effects on mTORC2 signalling, hepatic urea cycling, markers of neuroinflammation and sarcopenia [15–19]. Importantly, these male-specific benefits occur without major feminization of sex hormone profiles [10] or reproductive function [20]. Therefore, 17αE2 may have the potential as a future therapeutic for the intervention of age-related metabolic disease, without the risk profile and significant side effects normally associated with estrogen treatment.

Until recently, the receptor(s) that mediate the actions of 17αE2 were believed to be uncharacterised [21–24] due to the relatively low binding affinity of 17αE2 for classical estrogen receptors alpha and beta (ERα and ERβ) when compared to 17βE2 in some settings [25, 26]. However, a recent study has strongly implicated ERα in 17αE2-mediated metabolic benefits. This report showed that 17αE2 and 17βE2 elicited almost identical genomic actions through ERα in an in vitro system. The report clearly demonstrated that global ablation of ERα attenuated nearly all the metabolic benefits of 17αE2 in male mice on a high-fat diet (HFD), indicating that 17αE2 signals through ERα to elicit health benefits [12]. This study also provided evidence that the brain is one of the primary regions where 17αE2 signals modulate systemic metabolism, as central treatment with 17αE2 via intracerebroventricular delivery also significantly improved insulin sensitivity and glucose tolerance [12].

The arcuate nucleus (ARC) of the hypothalamus is a well-established regulator of body weight and food intake [27]. In response to 17αE2 treatment, ERα abundance has been shown to increase in the ARC, specifically in male mice, suggesting that 17αE2 is activating this receptor within this brain region [19]. However, a causal role of ERα at this site in mediating the health or metabolic responses to 17αE2 has yet to be established. A previous study has focused on the role of pro‐opiomelanocortin (POMC, a precursor polypeptide that regulates anorexigenic signalling [28]) neurons in regulating responses to 17αE2, and these neurons are found within the ARC. Animals with an almost complete knockdown of POMC within the ARC showed a blunted response to 17αE2, although still showed some metabolic improvements in response to this treatment [11]. This suggests that neurons within this region are at least partly involved in mediating metabolic responses to 17αE2.

ERα is a critical regulator of energy homeostasis and is abundantly expressed throughout the ARC [29]. ERα KO animals show many symptoms of metabolic dysfunction, including impaired glucose and insulin signalling, increased adiposity and decreased energy expenditure [30, 31]. Given the significant interest in 17αE2 as a potential lifespan-prolonging agent and that ERα is a known modulator of metabolic functions in males, it is imperative to further understand how this steroid-receptor pathway functions in the context of 17αE2 treatment and when metabolically perturbed by HFD treatment. Prolonged HFD feeding and obesity in mice lead to many alterations in neuronal signalling in the ARC, including increasing neuronal firing and altering leptin signalling in both POMC and AgRP (precursor peptide that regulates orexigenic signalling) neuronal populations [32–34], which could influence responses to treatment.

Within the hypothalamus, most neurons utilise glutamate or GABA for neurotransmission. ERα expression in excitatory glutamatergic (GLUT) and inhibitory GABAergic (GABA) neurons is essential for estrogen signalling, at least in female mice [35]. Male mice with ERα deleted specifically within glutamatergic neurons (Vglut2-ERαKO mice) have increased serum testosterone and seminal vesicle weights in comparison with WT controls, demonstrating a loss of negative feedback by the hypothalamic-pituitary-gonadal (HPG) axis. Male mice with ERα deleted in GABAergic neurons (Vgat-ERαKO) do not show this loss of negative feedback but exhibit impaired masculinized behaviour [36].

In this study, we used Cre-lox recombination to delete ERα from either GLUT or GABA neurons as previously reported [35, 36]. We tested the hypothesis that ERα expression in one of these neuronal populations would be essential for mediating metabolic responses to 17αE2 in male mice. The ARC shows a mixture of GLUT or GABA neurons and therefore deletion of ERα in either of these populations was expected to lead to a strong reduction in ERα expression in this region [37]. Mice were given either a HFD or an identical HFD containing 17αE2 for 10 weeks. We recorded body weight, glucose tolerance, fasting insulin and hepatic lipid levels and also characterised the knockout of ERα expression in each model.

## Methods

### Mutant animals

*Vglut2*-ires-Cre, *Vgat*-ires-Cre and *Esr1*^lox/lox^ mice were sourced from collaborators at the University of Otago who had previously used these animals to establish the reproductive phenotype of these conditional deletions in females [35]. *Vglut2*-ires-Cre and *Vgat*-ires-Cre lines have previously been extensively characterised, showing localised expression of Cre recombinase in glutamatergic and GABAergic neurons, respectively [38]. *Vglut2*-ires-Cre and *Vgat*-ires-Cre mice were separately crossed with *Esr1*^lox/lox^ mice to generate mutant *Vglut2/Vgat*-ires-Cre; *Esr1*^lox/lox^ (Vglut2/Vgat-ERαKO) and littermate controls. Mutant animals contained one copy of the Cre allele and were homozygous for ERα flox (Vglut2^Cre/+^; Esr1^lox/lox^ or Vgat^Cre/+^; Esr1^lox/lox^). Control animals for the Vgat comparison were either heterozygous for Vgat^Cre^ and did not contain ERα flox (Vgat^Cre/+^; Esr1^+/+^) or did not harbour Vgat^Cre^ allele but were homozygous for ERα flox (Vgat^+/+^; Esr1^lox/lox^). No differences were observed between these control genotypes, and so they were pooled for analysis. Control animals for the Vglut2 comparison were negative for Vglut^Cre^ and homozygous for ERα flox (Vglut2^+/+^; Esr1^lox/lox^).

## Animal husbandry

Animal procedures were approved by the animal ethics committee at the University of Otago. Male mice were housed in a temperature- (21 °C) and light-controlled environment on a 12:12 light-to-dark cycle and had ad libitum access to food and water. Prior to the experiment, mice were housed in single-sex groups (2–5 animals/cage) and were fed Teklad Global 18% Protein Rodent Diet #2918 (protein 18.6%; fat 6.2%; carbohydrate 44.2%). Male mice were 4–7 months of age at the time of the experiment.

Vglut2-ERαKO and Vgat-ERαKO mice were used in separate experiments. Within each genotype we randomly assigned mice, stratified by age and weight, to one of four treatment groups: control high fat diet (HFD), control HFD + 17αE2, conditional ERα-deletion (either Vglut2- or Vgat) HFD, conditional ERα-deletion HFD + 17αE2. For each experiment, the sample size was as follows: glutamatergic genotype: WT HFD (*n* = 10), WT HFD + 17αE2 (*n* = 10), Vglut2-ERαKO HFD (*n* = 10), Vglut2-ERαKO HFD + 17αE2 (*n* = 10); GABAergic genotype: WT HFD (*n* = 13), WT HFD + 17αE2 (*n* = 14), Vgat-ERαKO HFD (*n* = 13), Vgat-ERαKO HFD + 17αE2 (*n* = 14 at the beginning of the experiment, one animal died due to illness so data was not included, final sample size *n* = 13). Mice in HFD groups were fed a 45% high-fat diet (TestDiet 58V8, 35.8% CHO, 18.1% PRO, 46.1% FAT, semi-purified) from TestDiet (Richmond, IN). Mice in the HFD + 17αE2 groups were fed a matched high-fat diet containing 17αE2 (TestDiet 58V8 + 17αE2, 14.4 ppm; Steraloids, Newport, RI). This dose of 17αE2 has been previously shown to extend lifespan in male mice [6] and has been used in studies showing that the metabolic benefits of 17αE2 are dependent on ERα expression [12]. Both HFD and HFD + 17αE2 diets were provided ad libitum over the entire treatment period.

**Fig. 1:**
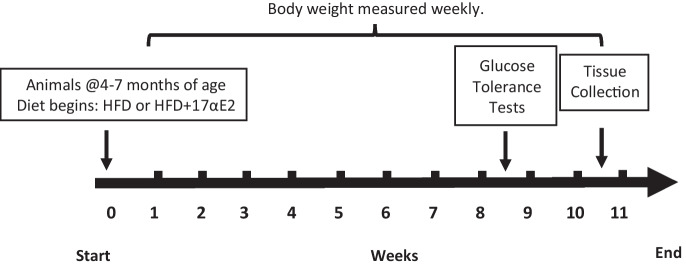
Schematic representation of experimental timeline. Animals were imported to the study between 4 and 7 months of age. Animals were assigned within genotype to treatment group, either HFD or HFD + 17αE2. Body weight was measured weekly for 10 weeks. Glucose tolerance testing was performed on weeks 8 and 9. Animals were sacrificed after 10 weeks for tissue collection

Mice were fed experimental diets for 10 weeks (Fig. 1). Over this period mice were weighed weekly. After mice had been on the experimental diets for 8–9 weeks, animals underwent a glucose tolerance test (Fig. 1). Two weeks later, mice were fasted for 6 h prior to euthanasia via cervical dislocation. A blood sample was collected for an insulin assay via cardiac puncture into an EDTA lined tube and left on ice, after which the sample was centrifuged at 1000g for 10 minutes and plasma was frozen at − 80 °C. The animals were dissected, tissue samples were collected and raw organ weights were recorded with a microbalance.

## Metabolic analyses

Mice were fasted for 6 h at 7–8 a.m. at the beginning of the dark period (water access remained ad libitum) prior to intraperitoneal (ip) injection 6 h later with 2 g/kg d-glucose (*n* = 10–14/group). Blood glucose levels were measured immediately pre-injection (time 0) and at 15-, 30-, 45-, 60-, 90-, and 120-min post-injection. Fasting insulin levels were assessed using plasma and the Mouse Ultrasensitive Insulin ELISA from Crystal Chem (*n* = 9–10/group) (Elk Grove Village, IL). Two values were higher than the highest value on the standard curve in the WT HFD group in the GABAergic genotype experiment, so the top value from the standard curve was used for each.

## Immunohistochemistry

Brains were dissected, post-fixed for 24 h in 4% paraformaldehyde in phosphate-buffered saline (PBS), then immersed in 30% sucrose in PBS for another 24 h before being frozen using liquid nitrogen and isopentane, embedded in OCT and stored at − 80 °C. Brains from both control and Vglut2/Vgat-ERαKO animals were coronally sectioned in a series of four at 30 µm using a cryostat and stored in cryoprotectant at − 20 °C prior to processing. Two medial ARC sections were anatomically matched from each animal and were treated with 30% hydrogen peroxide solution in methanol and Tris buffer saline (TBS) to inactivate any endogenous peroxidase activity prior to blocking (5% normal goat serum (NGS), TBS, 0.25% bovine serum albumin (BSA)). Sections were incubated for 24 h at 4 °C in a primary antibody against ERα (anti-rabbit, 1:10,000, Millipore Cat# 06–935, RRID:AB_310305) in an antibody dilution solution containing TBS with 2% NGS and 0.25% BSA. Sections were then incubated for 60 min at room temperature in a secondary biotinylated anti-rabbit immunoglobulin antibody (anti-rabbit, 1:500, Vector Laboratories Cat# BA-1000, RRID:AB_2313606) and then in Vectastain Elite Avidin–Biotin dissolved in antibody dilution solution (5 μl/ml solution, Vector Laboratories) for 90 min at room temperature. Benzene derivative DAB (3,3′-diaminobenzidine), nickel and glucose oxidase were used as a substrate to reveal a purple/brown derivative stain within the nucleus of positively stained cells for ERα.

## Immunohistochemistry analysis

The number of ERα positive cells was quantified in the ARC by counting the number of positively stained cells (*n* = 5–6/group). Two medial ARC sections from each mouse were analysed and the average count was used in the analysis. Sections were photographed using an Olympus 1501 microscope (10×). ERα-positive cells were counted automatically using ImageJ software with constant thresholding throughout the analysis. The investigator was blinded to the genotype and treatment group and conducted thresholding based on visible positive staining in the ARC. The ARC boundaries were defined consistently throughout the analysis. While blinded to treatment, a triangle to dictate the ARC region of interest (ROI) was drawn over a few animals, and then the same triangle which encompassed the best regional anatomy of the ARC for these animals was used for all animals in the study. We considered only the medial region of the ARC (Paxinos coordinates − 1.46 to − 1.70). Briefly, the ImageJ protocol was as follows: The standardised ROI was outlined in each image, the background was subtracted and the particle analysis was conducted with the following measurements: rolling ball radius set to 250, pixel size 100–infinity, circularity 0.25–1. Only ERα-positive cells with a stained nucleus were included in counts.

## Liver histology

Oil-red-O (ORO) staining was performed to assess lipid levels in liver samples as previously described (*n* = 8–9 per group) [39]. Twelve micron liver sections were sectioned at − 20 °C on a cryostat and stored at − 80 °C. ORO was dissolved in isopropyl to make a working solution, in which sections were incubated for 5 min. Sections were imaged within 24 h of staining on an Olympus 1501 microscope (10×). The red lipid stain was quantified on ImageJ software using 5 regions of interest per animal, and the investigator was blinded to genotype and treatment group. Staining is presented as a lipid-to-total tissue ratio. Thresholding was determined and remained consistent throughout the analysis.

## Statistical analyses

Statistics were performed using GraphPad Prism software (version 9) unless otherwise stated. Results for the two genotypes of mice were analysed separately. For each measured parameter, we conducted a two-way ANOVA, assessing the effect of 17αE2 treatment, the effect of genotype (either conditional knockout or control) and an interaction between genotype and treatment. An interaction between genotype and treatment would indicate that the genotypes respond differently to 17αE2 treatment. Where an interaction was present, we looked within each genotype to determine why the genotypes responded differently to treatment. Analysis of body weight changes was conducted using a Repeated Measures ANOVA in SPSS version 29, including body weight change from the start for each time point as a repeated dependent variable and genotype and treatment as independent variables. The results for tissue and organ weights are expressed in their raw values, i.e. not in relation to body weight. Results are presented as mean ± SEM, unless otherwise stated. The significant threshold for tests was α = 0.05.

## Results

### ERα positive cells in the arcuate nucleus are reduced in Vglut2-ERαKO and Vgat-ERαKO conditional knockout male animals

**Fig. 2:**
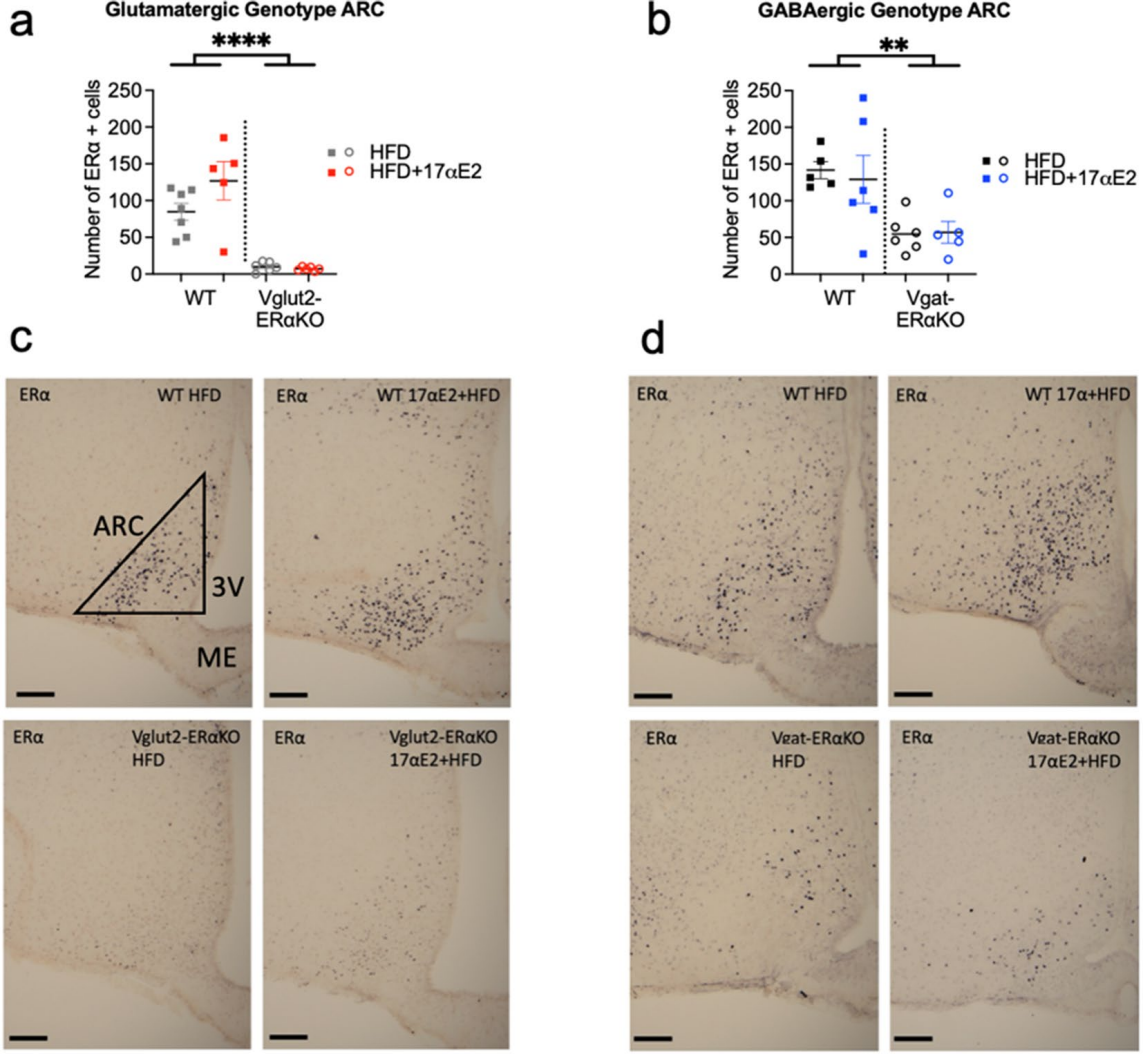
ERα expression is reduced in the ARC in Vglut2-ERαKO and Vgat-ERαKO male mice. Numbers of positive ERα cells in ARC in WT and Vglut2-ERαKO (**a**) or WT and Vgat-ERαKO (**b**) mice **P < 0.01, ****P < 0.0001 (two-way ANOVA, genotype and treatment as factors). Error bars show mean ± SEM. Representative images showing ERα is reduced in Vglut2-ERαKO (**c**) and Vgat-ERαKO (**d**) mice on both HFD and HFD + 17αE2 in comparison with WT controls. Scale bar = 100 µm (*n* = 5–6 per group). ARC, arcuate nucleus; 3V, third ventricle; ME, median eminence. Bregma coordinates − 1.46 to − 1.70 according to the Franklin and Paxinos mouse brain atlas

In order to validate the conditional ERα knockout in male mice, we quantified the number of ERα-expressing cells in the arcuate nucleus of the hypothalamus, an area that contains both glutamatergic and GABAergic neurons [37, 40], at the end of the study. In the glutamatergic genotype, Vglut2-ERαKO mice on both the HFD and HFD + 17αE2 diets showed a significant reduction in the number of ERα positive cells in the ARC in comparison to littermate controls (Fig. 2a, c) (overall effect of genotype *P* < 0.0001). There was no effect of 17αE2 treatment or interaction between genotype and treatment group on the number of ERα-positive cells. In the GABAergic genotype, Vgat-ERαKO mice also showed a significant reduction in the number of ERα positive cells in the ARC in comparison with WT controls (Fig. 2b, d) (overall effect of genotype *P* < 0.01). There was no effect of treatment or interaction between genotype and treatment. These results validate the successful knockout of ERα in both Vglut2-ERαKO and Vgat-ERαKO conditional knockouts.

### 17αE2 treatment reduces body weight and adiposity in WT, Vglut2-ERαKO and Vgat-ERαKO male mice

**Fig. 3:**
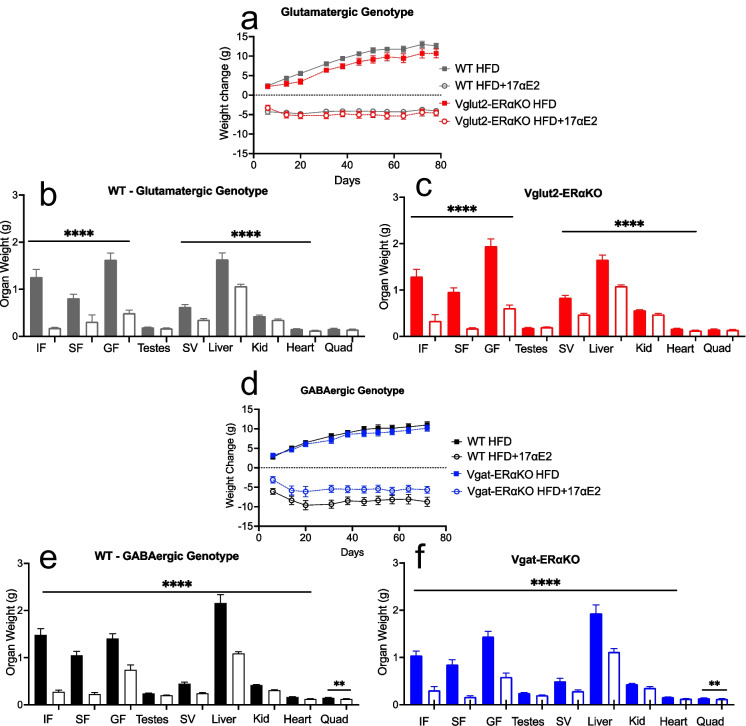
17αE2 treatment significantly reduces body weight and metabolically active organ weights across all genotypes. Body weight in WT and Vglut2-ERαKO groups receiving HFD or HFD + 17αE2 treatment (**a**). Fat pads, testes, seminal vesicles, liver, kidney, heart and quad weights in WT (**b**) and Vglut2-ERαKO (**c**) mice in response to HFD or HFD + 17αE2 treatment. Body weight in WT and Vgat-ERαKO groups receiving HFD or HFD + 17αE2 treatment (**d**). Fat pads, testes, seminal vesicles, liver, kidney, heart and quad weights in WT (**e**) and Vgat-ERαKO (**f**) mice in response to HFD or HFD + 17αE2 treatment. Bars above the lines in (**b**), (**c**), (**e**) and (**f)** represent organs where a significant main effect of 17αE2 was observed (also see Table 1) *P < 0.05, ****P < 0.0001 (from a two-way ANOVA for genotype or treatment factors). Error bars show mean ± SEM. (IF, inguinal fat; SF, subscapular fat; GF, gonadal fat; SV, seminal vesicles; Kid; kidney) (*n* = 10–14 per group)

At the start of the experiment, mice were placed either on a 45% HFD (TestDiet 58V8, 35.8% CHO, 18.1% PRO, 46.1% FAT) or on a matched high-fat diet containing 17αE2 (TestDiet 58V8 + 17αE2, 14.4 ppm). Within animals of the glutamatergic genotype comparison, almost immediately after initiation of 17αE2 treatment, animals began to lose a significant amount of body weight (Fig. 3a), which is consistent with previous findings [10, 11]. Both control and Vglut2-ERαKO mice lost a significant amount of weight over time in response to 17αE2 treatment (Fig. 3a). There was a main effect of 17αE2 treatment on body weight change over the experiment (main effect of 17αE2 treatment in a repeated measures ANOVA *P* < 0.001), but no interaction between genotype and treatment on body weight change (*P* > 0.1), suggesting deletion of ERα in glutamatergic neurons does not influence body weight responses to 17αE2. We did observe a main effect of genotype on body weight change over the experiment (main effect of genotype *P* < 0.05). Vglut2-ERαKO mice on a HFD gained less weight over time in comparison with their respective control animals. This suggests that loss of ERα in glutamatergic neurons may protect against weight gain on a HFD, although it does not influence the response to 17αE2.

At the end of the experiment, we examined the raw weight of a number of reproductive and metabolically active tissues. As expected from the body weight changes, 17αE2 significantly reduced the weights of fat pads of male mice (inguinal, subscapular and gonadal) in both WT and Vglut2-ERαKO animals (Fig. 3b, c; Table 1). No interaction between genotype and treatment was observed for the masses of these fat pads (Table 1), indicating that the reduction in adiposity with 17αE2 occurs largely independently from ERα expression in glutamatergic neurons. The only interaction between genotype and treatment that was observed was for testes weights (*P* = 0.0234), but there were no obvious genotype or treatment-specific trends which explained this, with testes mass slightly reduced with 17αE2 treatment in WT males and slightly increased in Vglut2-ERαKO males.

**Table 1:** *P* values for effects of 17αE2, genotype and interaction between 17αE2 treatment and genotype for organ weights in Vglut2-ERαKO and Vgat-ERαKO male mice at the end of the experiment

	Glutamatergic genotype			GABAergic genotype		
	Effect of 17αE2 (*P* value)	Effect of GT (*P* value)	Interaction (*P* value)	Effect of 17αE2 (*P* value)	Effect of GT (*P* value)	Interaction term (*P* value)
Inguinal fat	< 0.0001	0.6702	0.3728	< 0.0001	0.0259	0.0115
Subscapular Fat	< 0.0001	0.9453	0.1383	< 0.0001	0.0575	0.3185
Gonadal fat	< 0.0001	0.0644	0.3748	< 0.0001	0.5830	0.3639
Testes	0.2838	0.8488	0.0234	< 0.0001	0.6796	0.6366
Seminal vesicles	< 0.0001	0.0002	0.2445	< 0.0001	0.4918	0.7866
Kidney	< 0.0001	< 0.0001	0.7944	< 0.0001	0.0484	0.3382
Liver	< 0.0001	0.8423	0.9885	< 0.0001	0.4659	0.3356
Heart	< 0.0001	0.4322	0.6615	< 0.0001	0.7912	0.4972
Quad	0.1784	0.3932	0.8008	0.0039	0.4364	0.3660

In addition to the effects of 17αE2 on adipose tissue, the weights of the heart, liver, seminal vesicles and kidneys were also reduced by 17αE2 treatment in all genotypes of animals (Fig. 3b, c Table 1). These reductions also occurred largely independently of genotype and deletion of ERα in either genotype (Table 1). Seminal vesicle and kidney weight are very sensitive to circulating androgens and this reduction indicates that 17αE2 reduces certain aspects of androgen signalling as previously reported [17]. Interestingly, we observed that the seminal vesicles and kidneys were significantly heavier in the Vglut2-ERαKO animals in comparison with their WT controls (Fig. 3b, c Table 1). This is consistent with previous reports of Vglut2-ERαKO animals having heavier seminal vesicle weights and higher circulating testosterone levels [36] and suggests that ERα in glutamatergic neurons provides gonadal steroid hormone negative feedback signalling within the HPG axis in males.

Within the GABAergic genotype, both WT and Vgat-ERαKO animals on a HFD gained a significant amount of weight over the course of the experiment, while 17αE2 treatment reduced the body weight of both WT and Vgat-ERαKO animals (main effect of 17αE2 *P* < 0.001; Fig. 3d). A significant interaction between genotype and treatment was observed for body weight change over the experiment (interaction between genotype and treatment *P* < 0.05), which was due to Vgat-ERαKO animals losing less weight than WT animals on 17αE2 treatment (Fig. 3d), although these animals still responded strongly with substantial body weight loss to 17αE2. Assessing the weights of fat pads at the end of the experiment, there was no evidence of a main effect of genotype for subscapular or gonadal fat pad weight (Table 1). There was an interaction between genotype and treatment for inguinal fat pad weight (Table 1), with Vgat-ERαKO mice having lighter inguinal fat pads on a HFD than controls and the weight of this adipose tissue showing less of a change with 17αE2 (Fig. 3e, f), contributing to the diminished body weight loss response to 17αE2. The weights of all other measured organs were reduced with 17αE2 and this response was unaffected by genotype (Table 1).

### 17αE2 significantly improves glucose tolerance and reduces fasting insulin levels in both WT and Vglut2-ERαKO and Vgat-ERαko male mice

**Fig. 4:**
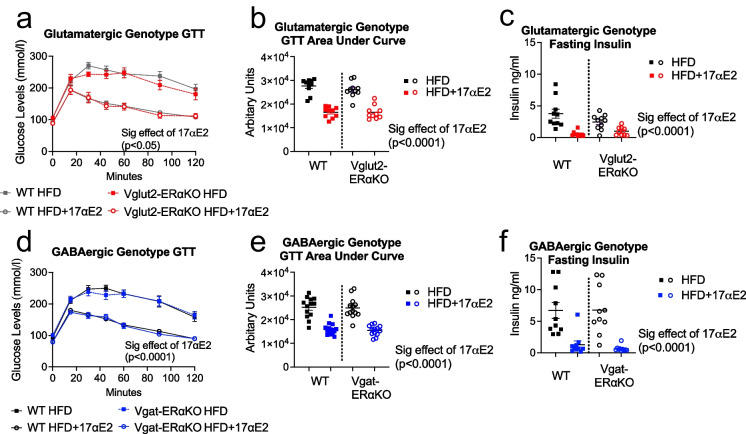
Glucose tolerance and fasting insulin levels are significantly improved in response to 17αE2 treatment across all genotypes. Glucose tolerance (**a**), area under the curve (**b**) and fasting insulin levels (**c**) in WT and Vglut2-ERαKO male mice in response to either a HFD or 17αE2 treatment on a HFD. Glucose tolerance (**d**), area under the curve (**e**) and fasting insulin levels **(f)** in WT and Vgat-ERαKO male mice in response to either a HFD or 17αE2 treatment on a HFD. Error bars show mean ± SEM. *P* values are from the main effect of 17αE2 treatment and are calculated from a two-way ANOVA (at each time point for glucose tolerance) including genotype and treatment as factors (*n* = 9–14 per group)

17αE2 treatment improves glucose tolerance and reduces fasting insulin levels in male mice [10, 15]. We conducted glucose tolerance tests and a fasting insulin assay to assess whether 17αE2 would provide these metabolic benefits when ERα is deleted in either glutamatergic or GABAergic neurons. 17αE2 strongly improved glucose tolerance, an effect that was independent of ERα deletion in either glutamatergic (Fig. 4a, b) or GABAergic neurons (Fig. 4d, e). In each comparison, there was a main effect of 17αE2 for glucose levels at each time point, and no difference between genotypes (i.e. between WT and Vglut2-ERαKO (Fig. 4a) or Vgat-ERαKO (Fig. 4d) mice) nor an interaction between genotype and treatment. This strong effect of 17αE2 but lack of genotype effect in each comparison is also illustrated in the area under the curve for glucose responses (Fig. 4b, e). Similarly, fasting plasma insulin concentrations were also reduced by 17αE2 independently of ERα in either genotype (Fig. 4c, f ). Collectively, these results show a significant capacity of 17αE2 to improve metabolic parameters in both WT and conditional ERα knockouts in the glutamatergic and GABAergic experimental groups.

### 17αE2 significantly reduces hepatic lipid levels in both WT and Vglut2-ERαKO and Vgat-ERαKO male mice

**Fig. 5:**
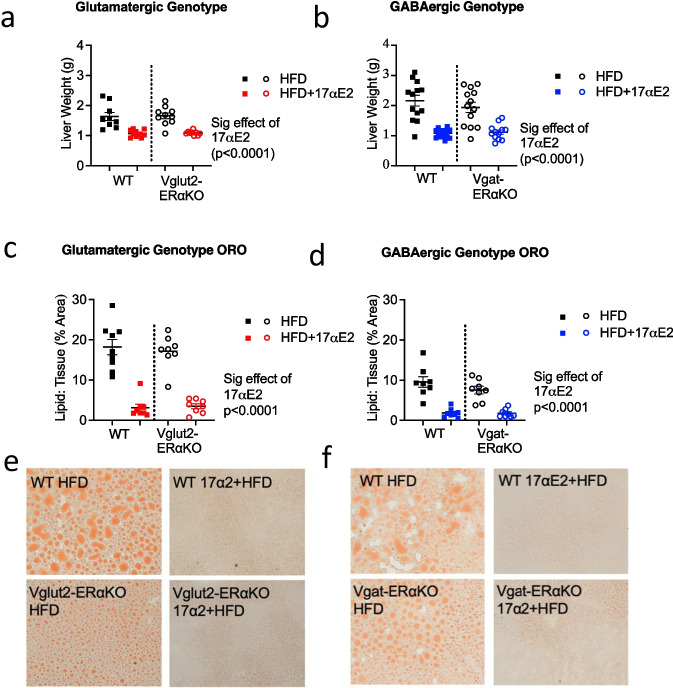
Liver weights and lipid levels are significantly reduced in response to 17αE2 treatment across all genotypes. Liver weights in WT and Vglut2-ERαKO (**a**) and in WT and Vgat-ERαKO mice (**b**) in response to either a HFD or 17αE2 treatment on a HFD. Liver lipid levels in WT and Vglut2-ERαKO (**c**) and in WT and Vgat-ERαKO (**d**) mice in response to either a HFD or 17αE2 treatment on a HFD. *P* < 0.0001 for the overall effect of 17αE2 treatment from a two-way ANOVA including genotype and treatment as factors. Error bars show mean ± SEM (*n* = 8–9 per group). Representative images showing lipid levels are reduced in response to 17αE2 treatment on a HFD in WT and Vglut2-ERαKO (**e**) and in WT and Vgat-ERαKO (**f**) mice

It has been previously shown that 17αE2 reduces lipid content in the livers of male mice in an ERα-dependant manner [12]. We observed that 17αE2 strongly reduced both liver weight (Fig. 5a, b) and hepatic lipid content (Fig. 5c-f) within all genotypes of mice in response to 17αE2 treatment. This illustrates that the effects of 17αE2 on hepatic adiposity do not depend on ERα in either glutamatergic and or GABAergic neurons.

## Discussion

The effects of 17αE2 on lifespan [6] and metabolic health [12, 15] occur in a sex-specific manner, preferentially benefiting male but not female mice. The physiology underlying this sexual dimorphism remains to be clearly elucidated, but a key role of ERα in mediating metabolic responses to 17αE2 has been established [12]. The ARC has been identified as a likely target of 17αE2 treatment because of its established role in the regulation of energy balance and metabolism [27]. We hypothesised that ERα expression in glutamatergic and/or GABAergic neurons, the principal excitatory and inhibitory neurons in the ARC and the whole brain, would be essential for 17αE2 to elicit positive effects on metabolism in males. Our findings do not support our hypothesis, with the deletion of ERα in either neuron type having little impact on metabolic responses to 17αE2 treatment, even though ERα abundance was reduced with the deletion of ERα in both genotypes of mice. This finding suggests that alternative neuronal populations, other CNS cell types or peripheral expression of ERα may be involved in mediating 17αE2 responses in males.

We measured ERα abundance in the ARC of all genotypes of mice in this study. Both Vglut2-ERαKO and Vgat-ERαKO animals exhibited a significant reduction in the expression of ERα in comparison with WT animals, independent of the treatment group. The ARC is a sexually dimorphic region with males having reduced numbers of dendritic spines and axosomatic synapses in comparison with females [41]. It has historically been thought that the ARC is principally GABAergic; however, recent reports have illustrated a mixed population of both glutamatergic and GABAergic neurons present within the ARC [37]. Our results highlight the presence of both neuronal populations within male mice, since both genetic manipulations led to a strong reduction of ERα in this region. It has previously been reported that 17αE2 significantly increases ERα abundance in the ARC of male mice, whereas in our study we found no consistent change in ERα abundance with 17αE2 treatment. The previous finding was reported in 2-year-old UM-HET3 mice (a genetically heterogenous mouse model used by the Interventions Testing Program for ageing studies [42]) that had been treated with 17αE2 for over a year and were not exposed to metabolic dysfunction via a HFD [19]. By contrast, our animals were less than a year old and had been exposed to HFD treatment for 11 weeks. One possibility for this discrepancy in results is that 17αE2 could have effects on ARC ERα abundance specifically in old animals, possibly slowing any decline in ERα expression that occurs within ageing. It is also possible that more subtle differences in ERα expression may be detected with 17αE2 treatment if a more thorough immunohistochemical assessment was conducted across the ARC or hypothalamus.

Body weight was significantly reduced in both the WT and Vglut2-ERαKO/Vgat-ERαKO animals treated with 17αE2, although this effect was slightly blunted in Vgat-ERαKO animals. This suggests that part of the body weight response to 17αE2 may depend on ERα expression in GABAergic neurons. A wide range of neurons across the brain express ERα and are GABAergic, including in the ARC, such as neuropeptide Y (NPY) neurons [35, 43]. GABAergic neurons in other brain regions also express ERα, including in the medial preoptic nucleus (MPN) and bed nucleus of the stria terminalis (BNST) [44]. It is possible that loss of ERα from one or several of these populations diminishes the response to 17αE2 due to an inability to elicit  a metabolic response via ERα signalling. It is also worth considering that ablation of ERα in these neurons may be indirectly interfering with other hormonal cues controlling metabolism. The hormone leptin’s role in driving anti-obesity effects is well established, by binding to leptin receptors on POMC (anorexigenic) neurons [38]. Leptin can also stimulate POMC anorexigenic pathways by disinhibition of NPY/GABA (orexigenic) neurons [45]. The knockdown of ERα expression in GABAergic neurons, such as, but not limited to, leptin receptor–expressing neurons, may influence these endocrine signalling pathways and play a role in the blunted body weight reduction in response to 17αE2 treatment.

In contrast, Vglut2-ERαKO animals on a HFD gained less weight than WT mice. This was unexpected as ERα is generally regarded as having an important protective role against metabolic dysfunction. Therefore, a blunted response to weight gain on a HFD in animals with an ERα ablation is counter to work conducted in global and brain-specific ERα mice, where the loss of ERα signalling causes mice to gain weight [30, 46]. One possible reason for this result may relate to Vglut2-ERαKO animals having increased seminal vesicle weights in comparison to WT controls, demonstrating disrupted negative feedback signalling of the HPG axis and elevated androgen signalling [36]. This increase in circulating androgens may increase reproductive investment and shift the energy balance to a more negative state, protecting against adiposity on a HFD.

Altogether, our results in Vglut2-ERαKO and Vgat-ERαKO animals suggest that while 17αE2 can significantly reduce body weight in a manner that is largely independent of ERα in glutamatergic or GABAergic neurons, these conditional knockouts do elicit slightly different phenotypes in response to both HFD and HFD + 17αE2 treatment. Understanding how ERα influences body weight changes in male mice, irrespective of 17αE2 and particularly whether responses differ between males and females, could be an important avenue for future research in terms of understanding the role of ERα in metabolic regulation. However, glucose tolerance, fasting insulin and liver triglyceride levels were all significantly improved following 17αE2 treatment in both WT and Vglut2-ERαKO/Vgat-ERαKO animals, without any evidence of a different response to 17αE2 in mice with reduced ERα expression. Collectively, these metabolic results illustrate that 17αE2’s protective metabolic effects are not mediated exclusively by ERα expression in glutamatergic or GABAergic neurons.

There are several possible alternative pathways that may mediate the responses to 17αE2 via ERα and would explain why the loss of expression in either glutamatergic or GABAergic neurons does not influence responses. It is possible that compensatory feedback mechanisms occur in Vglut2-ERαKO and Vgat-ERαKO models which accommodate for the loss of ERα in these neurons and maintain responses to 17αE2. It is also possible that other neuronal populations mediate the responses to 17αE2, or deletion in both populations simultaneously is required to inhibit responses. Alternatively, non-neuronal modulation may be central to 17αE2’s protective metabolic effects. Cell types such as microglia or astrocytes may be involved in mediating 17αE2 effects on metabolism. As 17αE2 has previously been shown to reduce microglial numbers in the ARC in male mice [19], it is possible that non-neuronal mechanisms could play a significant role in modulating 17αE2s actions at the level of the brain. It is also quite plausible that peripheral estrogen receptors, acting either independently or additively with those found in the CNS, may be required to elicit systemic improvements in metabolism. Tissue-specific ERα knockouts, particularly in reproductive and metabolically active tissues, will help to elucidate control of 17αE2 at peripheral and central levels.

To conclude, our results indicate that 17αE2’s protective metabolic effects are not mediated exclusively by ERα expression in glutamatergic or GABAergic neurons. We show 17αE2 significantly improved several metabolic parameters, despite ERα deletion from glutamatergic or GABAergic neurons. Future studies targeting central and peripheral regulation of 17αE2 responses via ERα are required to further elucidate the mechanistic actions of 17αE2 in perpetuating positive effects on metabolism in males.
